# MRI Analysis: Optimization of parameters for diffusion MRI to enhance hippocampal subfield analysis and segmentation (Preliminary Data)

**DOI:** 10.1192/j.eurpsy.2022.1637

**Published:** 2022-09-01

**Authors:** P. Nwaubani, A. Colasanti, M. Cercignani, A. Warner

**Affiliations:** Brighton and Sussex Medical School (BSMS), Clinical Neuroimaging And Neuroscience, Brighton, East Sussex, United Kingdom

**Keywords:** Hippocampus, Neuroimaging, IQT, Neuroscience

## Abstract

**Introduction:**

The hippocampus is an important, complex limbic structure anatomically embedded in the medial temporal lobe of each cerebral cortex, which has been implicated in the pathogenesis of neuro-inflammatory disease conditions. Few studies have focused on the characterization of the MRI neuroimaging signatures of highly physio- pathologically relevant subfields of the hippocampus (CA1, CA4-DG, CA2/CA3, SLRM).

**Objectives:**

Using self-guided manually segmented, Diffusion weighted and NODDI maps created from data obtained from the Human Connectome Project (HCP) we intend to test whether Diffusion MRI-based quantitative imaging parameters (MD, FA, ODI, ISOVF, ICVF), indicative of microstructural characteristics of major hippocampal subfields (CA1, CA2/CA3, CA4-DG and SLRM), correspond to predictions for animal literature and imaging-histology correlations. We will also explore the correlations between these parameters and age.

**Methods:**

We used images from the Public connectome data (updated April 2018), exploring subjects with the 3T MRI sessions obtainable from the WU-Minn HCP Data section. For the purpose of this study, we selected and downloaded 10 preliminary imaging data (6 females and 4 males) based on age variability in the following ranges (26-30, 31-35 and 36+). We manually segmented, and computed quantitative parameters.

**Results:**

Converging and consistent literature allude to decreasing volumes with increasing age. Analyzing the volumes from the diffusion maps (pilot data), this was also the case, with volumes computed from CA1 and DG-CA4 sub regions. IQT also allowed for better appreciation of neuroanatomical boundaries and land marks, hence allowing more regions to be easily manually segmented (addition of CA2/CA3).

**Conclusions:**

Application to Neuroinflammatory imaging data.

**Disclosure:**

No significant relationships.

